# The diagnostic value of the ocular tilt reaction plus head tilt subjective visual vertical (±45°) in patients with acute central vascular vertigo

**DOI:** 10.3389/fneur.2022.1022362

**Published:** 2022-11-29

**Authors:** Yufei Feng, Tongtong Zhao, Yuexia Wu, Xia Ling, Menglu Zhang, Ning Song, Ji-Soo Kim, Xu Yang

**Affiliations:** ^1^Department of Neurology, Aerospace Center Hospital, Peking University Aerospace School of Clinical Medicine, Beijing, China; ^2^Department of Neurology, The First Affiliated Hospital of Jinzhou Medical University, Jinzhou, China; ^3^Department of Neurology, Seoul National University College of Medicine, Seoul, South Korea; ^4^Dizziness Center, Seoul National University Bundang Hospital, Seongnam, South Korea

**Keywords:** central vascular vertigo, ocular tilt reaction, subjective visual vertical, skew deviation, A/E-effect, unilateral peripheral vestibular disorders

## Abstract

**Objectives:**

To investigate the localization diagnostic value of the ocular tilt reaction (OTR) plus head tilt subjective visual vertical (SVV) in patients with acute central vascular vertigo (ACVV).

**Methods:**

We enrolled 40 patients with acute infarction, 20 with unilateral brainstem infarction (BI) and 20 with unilateral cerebellar infarction (CI). We also included 20 patients with unilateral peripheral vestibular disorders (UPVD) as the control group. The participants completed the OTR and SVV during head tilt (±45°) within 1 week of symptom onset.

**Results:**

In patients with ACVV, including that caused by lateral medullary infarction (100%, 2/2), partial pontine infarction (21%, 3/14), and cerebellum infarction (35%, 7/20), we observed ipsiversive OTR, similar to that seen in UPVD patients (80.0%, 16/20). Some of the patients with medial medullary infarction (50%, 1/2), partial pons infarction (42%, 6/14), midbrain infarction (100%, 2/2), and partial cerebellum infarction (30.0%, 6/20) showed contraversive OTR. The skew deviation (SD) of the BI group with ACVV was significantly greater than that of the UPVD group (6.60 ± 2.70° vs. 1.80 ± 1.30°, *Z* = −2.50, *P* = 0.012), such that the mean SD of the patients with a pons infarction was 9.50° and that of patients with medulla infarction was 5.00°. In ACVV patients with no cerebellar damage, the area under the curve of the receiver operating characteristic curve corresponding to the use of SD to predict brainstem damage was 0.92 (95%CI: 0.73–1.00), with a sensitivity of 100% and a specificity of 80% when SD ≥ 3°. We found no statistical difference in SD between the UPVD and CI groups (1.33 ± 0.58° vs. 1.80 ± 1.30°, *Z* = −0.344, *P* = 0.73). Compared with the UPVD patients, the ACVV patients with a partial pons infarction (43%, 6/14, χ^2^ = 13.68, *P* = 0.002) or medulla infarction (25%, 1/4, χ^2^ = 4.94, *P* = 0.103) exhibited signs of the ipsiversive E-effect with the contraversive A-effect, while those with a partial medulla infarction (50%, 2/4), pons infarction (43%, 6/14), or cerebellar infarction (60%, 12/20) exhibited a pathological symmetrical increase in the E-effect.

**Conclusions:**

The evaluation of OTR plus head tilt SVV (±45°) in vertigo patients is helpful for identifying and diagnosing ACVV, especially when SD is ≥ 3° or the E-effect is symmetrically increased.

## Introduction

Clinically, the incidence of dizziness/vertigo is high (1). According to population-based questionnaires, about 20–30% of people have experienced dizziness/vertigo (2). Studies have shown that at least 4 million patients in the United States visit the emergency department for acute dizziness/vertigo each year (3). Of these, about 1 million patients are routinely overtested to exclude malignant events such as stroke, even though one-third of strokes remain misdiagnosed (4, 5). The early and accurate identification of acute central vascular vertigo (ACVV) is thus an important issue for clinicians.

In recent years, with the rapid development of vestibular science, including clinical theories and related evaluation methods, it has become increasingly possible to accurately locate and diagnose vertigo/vestibular diseases. Studies have shown that evaluations based on the function of the otolith pathway, such as the ocular tilt reaction (OTR) test, are of great value in localizing and diagnosing vertigo-related issues affecting the central and peripheral areas (6). The OTR test includes head tilt (HT), skew deviation (SD), ocular torsion (OT), and subjective visual vertical (SVV) tilt. In previous studies, the rates of abnormal HT, SD, abnormal OT, and abnormal SVV in patients with acute peripheral vestibular syndrome were 4–20%, 14–29%, 19–82%, and 50.6–94% (7–11), respectively. The rates of abnormal HT, SD, abnormal OT, and abnormal SVV in ACVV patients were 3–38%, 29–31%, 57–83%, and 74.1–94% (12–14), respectively. The SVV is known to be the most sensitive index in the OTR. Although the degree of SD in the OTR has been found to be important in recent years, especially in the differentiation of peripheral and central diseases, its diagnostic value in assessing cerebellar damage is unclear. Furthermore, the prevalence of SD is low in both patients with peripheral and central vestibular disorders (12, 15). Korda et al. showed that the incidence of SD was 24% in patients with acute vestibular syndrome and 29% in those with stroke and that an SD > 3.3° had a high diagnostic value in identifying ACVV.

Head tilt SVV has also been found to be helpful in the localization and diagnosis of vertigo/vestibular diseases (16). However, whether it can be combined with the OTR to improve the localization and lateral diagnosis of peripheral and central damage has not been established. To address this, we assessed the diagnostic value of the traditional OTR plus head tilt SVV (±45°) in ACVV patients, including those with brainstem infarction (BI) and those with cerebellum infarction (CI). Our goal was to provide clinical evidence to facilitate the accurate diagnosis of ACVV.

## Materials and methods

### Participants and protocol

A prospective, cross-sectional study design was implemented in a tertiary hospital with an advanced vertigo center from April 8, 2021 to May 20, 2022. The target population was patients presenting for acute dizziness or vertigo. The inclusion criteria were: 1) Acute dizziness or vertigo; 2) Continuous dizziness or vertigo at the time of examination; 3) Age ≥ 18 years. The exclusion criteria were: 1) Dizziness or vertigo attributed to head trauma, orthostatic hypotension, or a known medical or neurologic disorder (e.g., hepatic encephalopathy, hydrocephalus); 2) Benign paroxysmal positional vertigo; 3) Severe new-onset with large lesions in MRI which could not cooperate with SVV and OTR tests; 4) Not cooperative to complete brain MRI; 5) Ophthalmoplegia which caused by lesions involving the oculomotor or trochlear nucleus, oblique neck, scoliosis or pelvic tilt that may affect the results of OTR/ head tilt SVV evaluation; 6) Incomplete data. Forty patients with ACVV (33 men, mean age 59.32, range: 38–75 years old) were finally enrolled (20 patients with acute unilateral BI and 20 patients with acute unilateral CI). Twenty patients with unilateral peripheral vestibular disorders (UPVD) (13 men, mean age 51.8, range: 28–66 years old) were also included as a control group. We also recruited 30 healthy subjects (16 men, mean age, 31.9 years old, range 22–63 years old) as the healthy control group. The diagnosis of BI and CI were confirmed by diffusion-weighted MRIs with an onset time ≤7 days. The diagnosis of UPVD was based on caloric canal paresis (CP) > 25%, indicating unilateral horizontal semicircular canal hypofunction, and onset time of spontaneous vertigo ≤7 days. All evaluations of OTR/ head tilt SVV (±45°) were performed during the acute phase (within 7 days from the symptom onset) with a mean interval of 4 days. To ensure the safety and reliability of the assessment, OTR/ head tilt SVV (±45°) were evaluated by two experienced neurologists. The patient sat in the back of a chair seat (in the outpatient clinic or ward). One examiner helped the patient maintain the appropriate head position, and the other evaluated the patient in the order of HT, SD, head upright SVV, head tilt SVV (±45°), and OT. The time required to complete the above assessments is 15–25 min.

This study was approved by the Ethics Committee of Peking University Aerospace Clinic School of Medicine and was conducted in accordance with the Declaration of Helsinki.

### HT

Before measurement, the patient was required to keep the body and head straight as far as possible while the examiner observed the patient's posture. If the height of the patient's shoulders was not at the same level, the patient was verbally prompted to adjust the height of the shoulders to keep the shoulders at the same level. After the adjustment, the examiner measured the angle between the sagittal axis of the patient's head and gravity with the protractor of the iPhone, which was the degree of head tilt. HT > 2° was defined abnormal (9).

### OT

Fundus photography was performed using a Nonmyd α-DIII retinal camera (Kowa American Corporation). Before capturing the image, the patient was instructed to adapt to the darkroom for 5 min to ensure their pupils were enlarged. Their head was required to be completely upright, and fixation was required while capturing the image. After photographing one eye, the patient was instructed to rest for 3 min with his or her eyes closed. After pupil recovery, the other eye was photographed in the same way. Ocular torsion was determined by measuring the angle formed by a horizontal meridian running through the center of the disc and a straight line passing through the center of the disc and the fovea. A difference in the torsional degree between the two eyes ≥ 8.8° was considered abnormal (12).

### SD

A Maddox rod was placed in front of the patient's right eye, and they were asked to focus both eyes on a light source located 33 cm away.

If SD was absent, the points and lines were seen to coincide; If an SD was presented, it was abnormal, and the patients could detect the separation of the points and lines. When separation was detected, we capped the prism for quantification. When the point and the line of separation overlap, the degree of the prism was taken as the patient's SD (15).

### SVV

The SVV was measured using VertiSVV (ZT-SVV-I, Shanghai ZEHNIT Medical Technology Co., Ltd., Shanghai, China). The system consists of a pair of Virtual Reality (VR) goggles, a wireless controller, and a laptop computer. The VR goggles display a luminous line in a completely dark background without visual cues. The VR goggles created a black visual field, and a yellow light bar with a length of 60 cm was projected into this field 2 meters away. The initial position was set to within ±25° (0° reflects the direction of gravity), and the participant adjusted the line's orientation by turning the knob of the wireless controller clockwise or counter-clockwise. Once the participant judges the luminous line to be aligned with true vertical, he or she confirms the position by pressing the confirm button on the wireless controller. The SVV angle and the head position of the subject were then recorded by the PC software (VertiPACS, ZEHNIT, Shanghai, China). We measured SVV in the head upright position, the head tilted to the right ear down 45° position (+45°), and the head tilted to the left ear down 45° position (−45°). The precision of the wireless controller was ± 0.1°. The examiner fixed the patient's head with both hands to complete the SVV measurements from all angles and controlled the change amplitude of the patient's head within ±1°. Each subject completed nine adjustments of the luminous line in each head position. After the subjects practiced the test twice, the test values were recorded and saved during the third trial to eliminate the influence of visual memory. The SVV adjustments were finally retained 7 times and averaged. In the healthy controls, the SVV was defined as positive when the tilt was rightward from the participant's perspective. However, to find the consistency between the SVV tilt side and the lesion side from the results of patients with left- and right-sided lesions, this paper specifies that positive SVV equates to roll-tilt toward the affected side (“ipsilesional”). In contrast, negative SVV is toward the patients' healthy side (“contralesional”). The head-upright SVV (HU-SVV) exceeding the range (−2.5 to +2.5°) was defined abnormal (17). We obtained the head tilt SVV(HT-SVV) errors by subtracting the SVV in the head upright position from that in the head tilted (±45°) positions. In both controls and patients, errors in HT-SVV were defined as negative values when the shift was in the opposite direction of head tilt, indicating the E-effect. Likewise, the errors in HT-SVV were defined as positive values when the shift induced by a head tilt in the same direction, indicating the A-effect (18). OTR was defined positive if a patient had any component of OTR (HT, SD, OT, and SVV).

### Statistical analyzes

Statistical analyzes were performed using SPSS software (version 25.0, IBM SPSS Statistics, N.Y., USA). Continuous variables were expressed as the mean ± SD. Categorical variables were expressed as percentages. Data normality was determined using the Shapiro-Wilk test. We used an independent sample *t*-test or nonparametric Mann-Whitney test to compare the groups. The Chi-square (χ^2^) test was also used for group comparisons, and Yates' continuity correction or Fisher's exact test was performed if necessary. All data were tested using two-sided tests, and *P* < 0.05 was considered statistically significant.

## Results

### Prevalence and lateralization of the OTR

**Figure 1:**
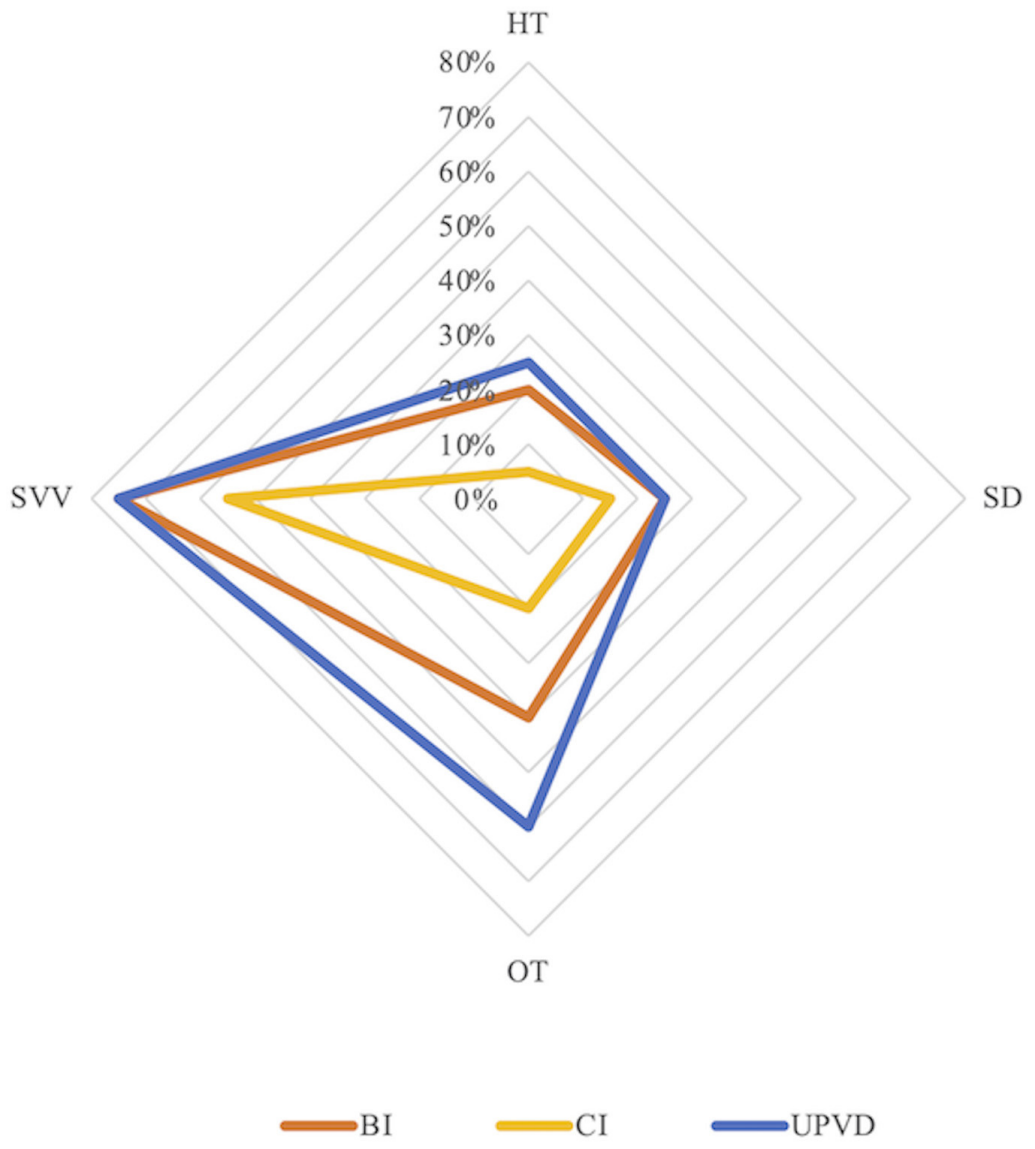
Prevalence of skew deviation (SD), ocular torsion (OT), head tilt (HT), and subjective visual vertical (SVV) tilt in the three groups. BI, brainstem infarction; CI, cerebellum infarction; UPVD, unilateral peripheral vestibular disorders.

The prevalence of abnormal HT, SD, abnormal OT, and abnormal HU-SVV tilt was 20.0, 25.0, 40.0, and 70.0%, respectively, in the BI group, 5.0, 15.0%, 20.0, and 55.0%, respectively, in the CI group, 25.0, 25.0, 60.0, and 75.0%, respectively, in the UPVD group (Table 1). The prevalence of abnormal OT in the UPVD group was significantly higher than in the CI group (60.0 vs. 20.0%, χ^2^ = 6.997, *P* = 0.042, Pearson Chi-square test). The prevalence of abnormal HT, SD, and abnormal HU-SVV tilt was not significantly different among the three groups (*P* > 0.05, Pearson Chi-square test, Figure 1).

**Table 1:** Prevalence of the components of OTR in three groups patients.

	**BI(*n* = 20)**	**CI(*n* = 20)**	**UPVD(*n* = 20)**	***P*** ^*^
Abnormal HT	20.0% (4/20)	5.0%(1/20)	25.0%(5/20)	0.305
SD	25.0%(5/20)	15.0%(3/20)	25.0%(5/20)	0.789
Abnormal OT	40.0%(8/20)	20.0%(4/20)	60.0%(12/20)	0.042
Abnormal HU-SVV	70.0%(14/20)	55.0%(11/20)	75.0%(15/20)	0.481
Ipsiversive OTR	25.0%(5/20)	35%(7/20)	80%(16/20)	0.001
Contraversive OTR	45.0%(9/20)	30%(6/20)	5%(1/20)	0.011

BI, brainstem infarction; CI, cerebellum infarction; HT, head tilt; HU-SVV, head upright subjective visual vertical; OT, ocular torsion; OTR, ocular tilt reaction; SD, skew deviation; UPVD, unilateral peripheral vestibular disorders.

* based on Chi-square test.

**Figure 2:**
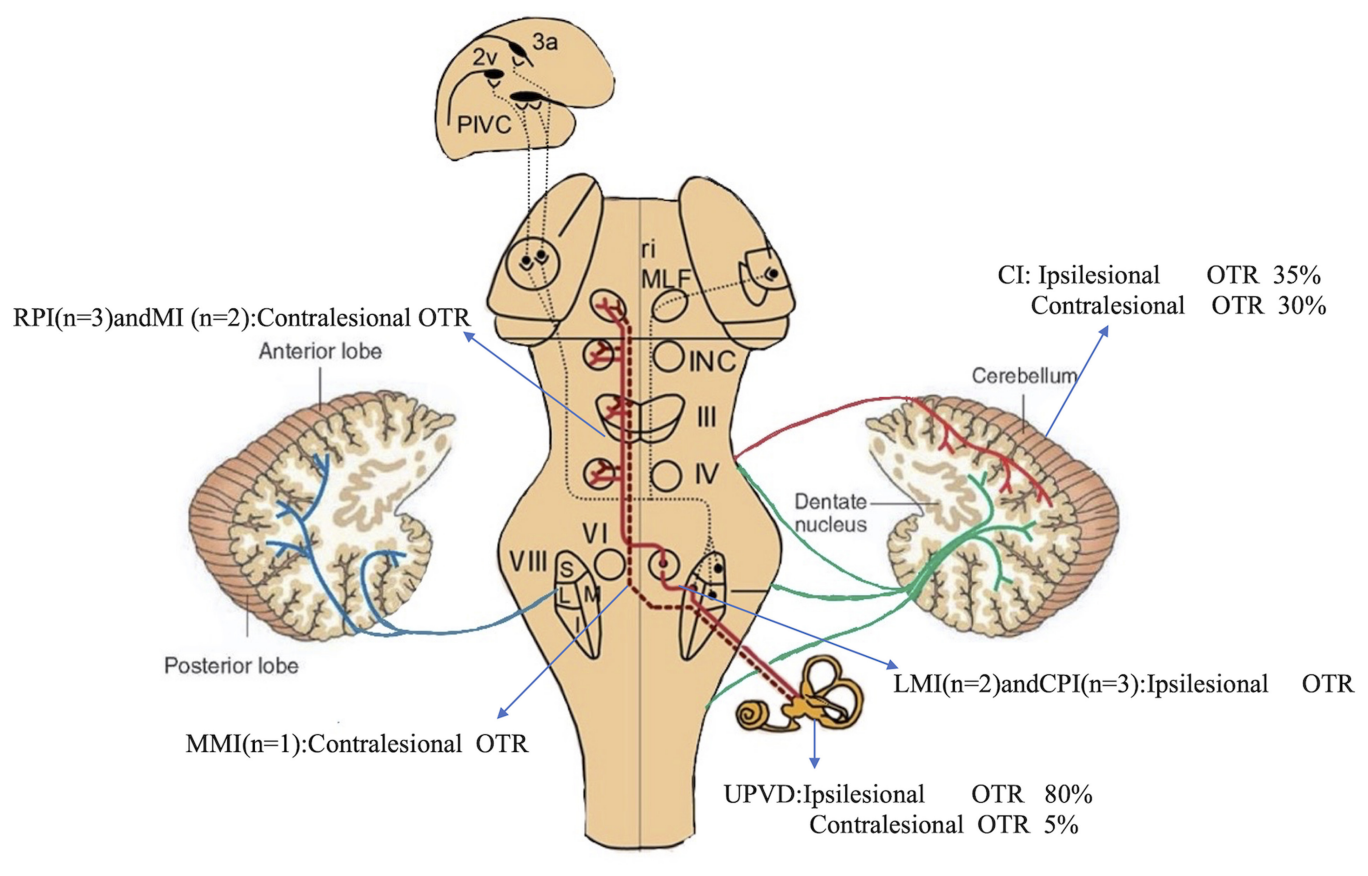
Incidence and direction of pathological OTR caused by different structural injuries. Pathways from the utricles and vertical semicircular canals mediate graviceptive function in the frontal roll plane. These pathways ascend from the vestibular nuclei (VIII) to the ocular motor nuclei, including the trochlear nucleus (IV), oculomotor nucleus (III), and abducens nucleus (VI). From here, they travel to the supranuclear centers of the interstitial nucleus of Cajal (INC), and the rostral interstitial nucleus of the medial longitudinal fasciculus (riMLF) in the midbrain tegmentum. RPI, rostral pontine infarction; CPI, caudal pontine infarction; MI, midbrain infarction; blue line, vestibulocerebellar fibers; green line, reticulocerebellar fibers; red line, pontocerebellar fibers [modified from Dieterich and Brandt (19)].

OTR was ipsiversive in 25% (5/20) of the patients with BI (lateral medullary infarction (LMI) 2, pontine infarction 3), 35% (7/20) of the patients with CI (tonsil and biventer lobule 5, biventer lobule and inferior semilunar lobule 1, cerebellar middle peduncles 1), and in 80% (16/20) of the patients with UPVD. OTR was contraversive in 45% (9/20) of the patients with BI (medial medullary infarction (MMI) 1, pontine infarction 6, midbrain infarction 2), 30% (6/20) of the patients with CI (tonsils, biventer lobule, and inferior semilunar lobules 2, posterior paravermis 1, dentate nucleus 2, nodulus 1), and in 5% (1/20) of the patients with UPVD (Figure 2). The direction of the OTR was significantly correlated with the lesion side (Kappa = 0.862, *P* < 0.05, McNemar test).

### Localization of the OTR

**Figure 3:**
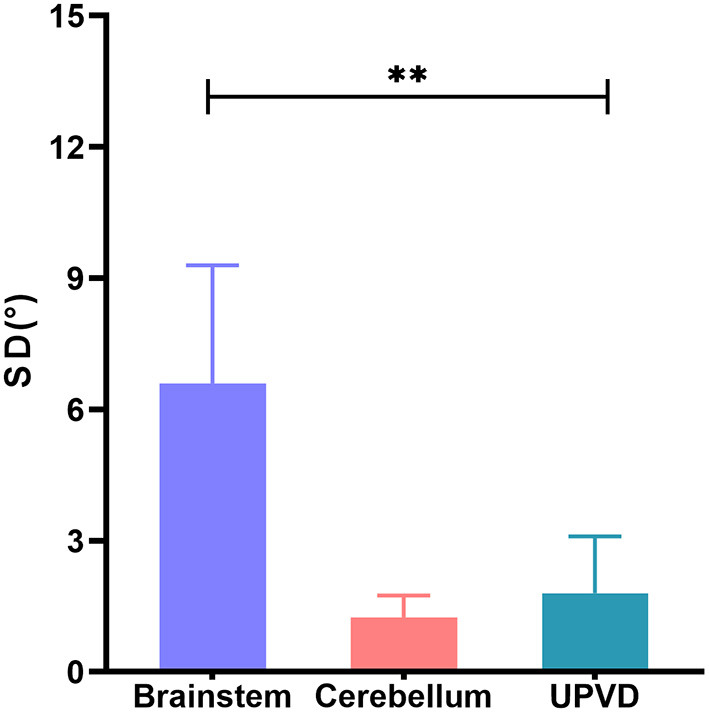
Comparison of SD of brainstem, cerebellum, and UPVD groups. SD, skew deviation; UPVD, unilateral peripheral vestibular disorders. **p < 0.05.

**Figure 4:**
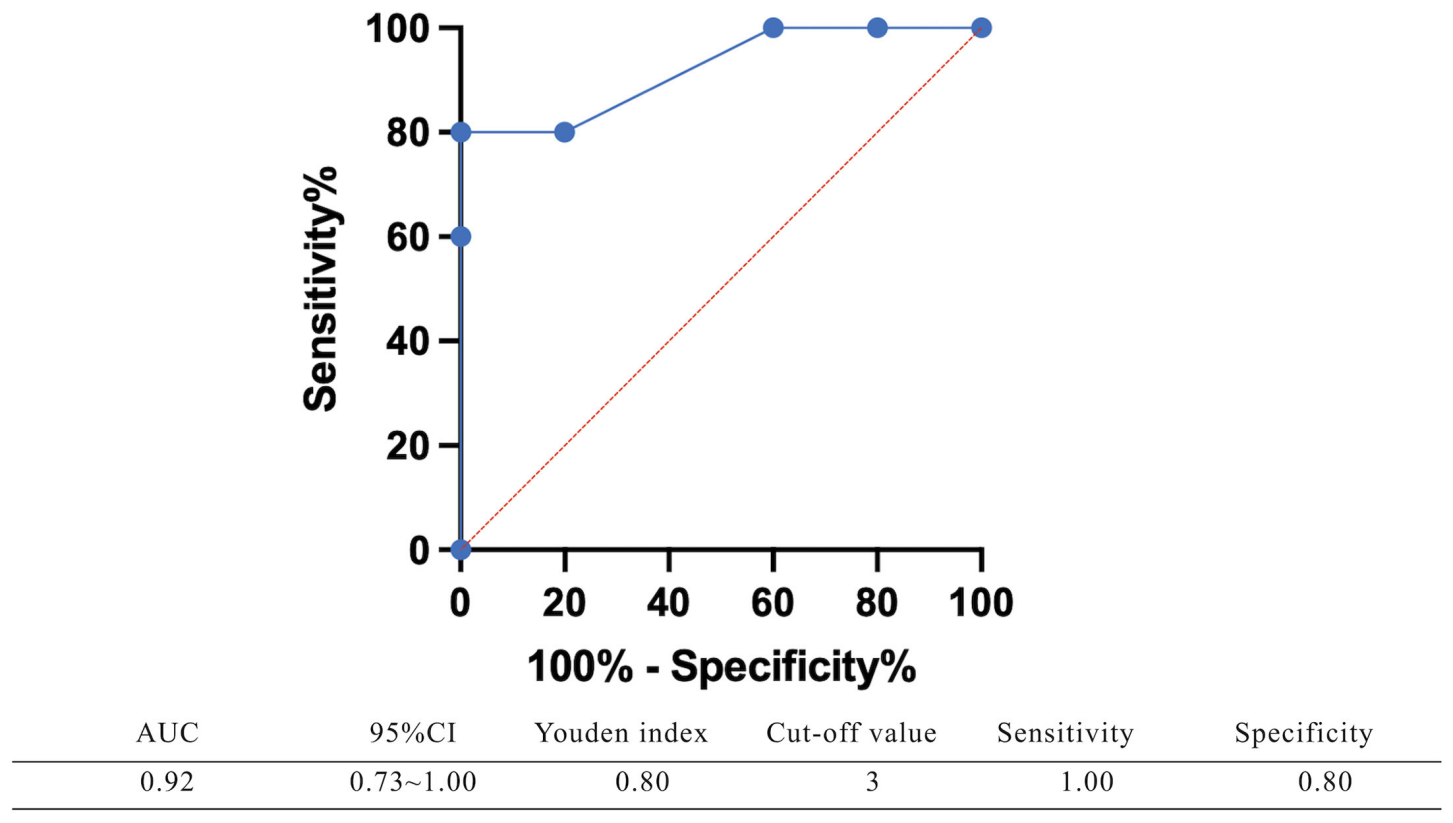
The receiver operating characteristic (ROC) curve of using SD predicting brainstem damage. The area under the curve (AUC) of the receiver operating characteristic curve was 0.92 (95%CI: 0.73–1.00), with a sensitivity of 100% and a specificity of 80% when SD ≥ 3°.

The SD in the BI group was significantly greater than that in the UPVD group (6.6 ± 2.7° vs. 1.8 ± 1.3°, *Z* = −2.50, *P* = 0.012, Mann-Whitney test). Further, the mean SD of the pontine infarction in the BI group was 9.5°, and that of the medullary infarction was 5.0°, which was significantly greater than that in the UPVD group (*Z* = −2.10, *P* = 0.044; *Z* = −2.03, *P* = 0.042, Mann-Whitney test). We found no statistical difference in SD between the UPVD and CI groups (1.3 ± 0.6° vs. 1.8 ± 1.3°, *Z* = −0.344, *P* = 0.73; Figure 3). Furthermore, the OT in the CI group was significantly smaller than that in the UPVD group (10.3 ± 10.4° vs. 5.6 ± 7.4°, *Z* = −2.93, *P* = 0.03, Mann-Whitney test), but the OT was not statistically different between the BI and the UPVD groups (10.3 ± 10.4° vs. 12.6 ± 9.7°, *Z* = −1.011, *P* = 0.312, Mann-Whitney test) and between the BI and the CI groups (10.3 ± 10.4° vs. 5.6 ± 7.4°, *Z* = −0.624, *P* = 0.533, Mann-Whitney test). There were no statistical differences in HT (5.1 ± 1.7° vs. 5.3 ± 1.9° vs. 4.0°, *H* = 1.580, *P* = 0.450, Kruskal-Wallis H test) or the degree of HU-SVV tilt (4.6 ± 5.5° vs. 3.1 ± 3.0° vs. 6.7 ± 5.7 °, *F* = 1.17, *P* = 0.318, one-way ANOVA) among the three groups (Table 2). To evaluate the ability of SD to predict BI, we constructed receiver operating characteristic (ROC) curves and calculated the area under the curve (AUC) (Figure 4).

**Table 2:** Test results of components of OTR in three patient groups.

	**BI**	**CI**	**UPVD**	***p***
HT	5.1 ± 1.7°(*n* = 4)	4.0°(*n* = 1)	5.3 ± 1.9°(*n* =5)	0.450^a^
SD	6.6 ± 2.7°(*n* = 5)	1.3 ± 0.6°(*n* = 3)	1.8 ± 1.3°(*n* = 5)	0.012^b^
OT	10.3 ± 10.4°(*n* = 20)	5.6 ± 7.4°(*n* = 20)	12.6 ± 9.7°(*n* = 20)	0.042^b^
HU-SVV	4.6 ± 5.5°(*n* = 20)	3.1 ± 3.0°(*n* = 20)	6.7 ± 5.7°(*n* = 20)	0.318^c^

BI, brainstem infarction; CI, cerebellum infarction; HT, head tilt; HU- SVV, head upright subjective visual vertical; n, number of patients; OT, ocular torsion; SD, skew deviation; UPVD, unilateral peripheral vestibular disorders.

a based on Kruskal-Wallis H test,

b based on Mann-Whitney test, and

c based on one-way ANOVA.

### HT- SVV and E-effect/A-effect

**Figure 5:**
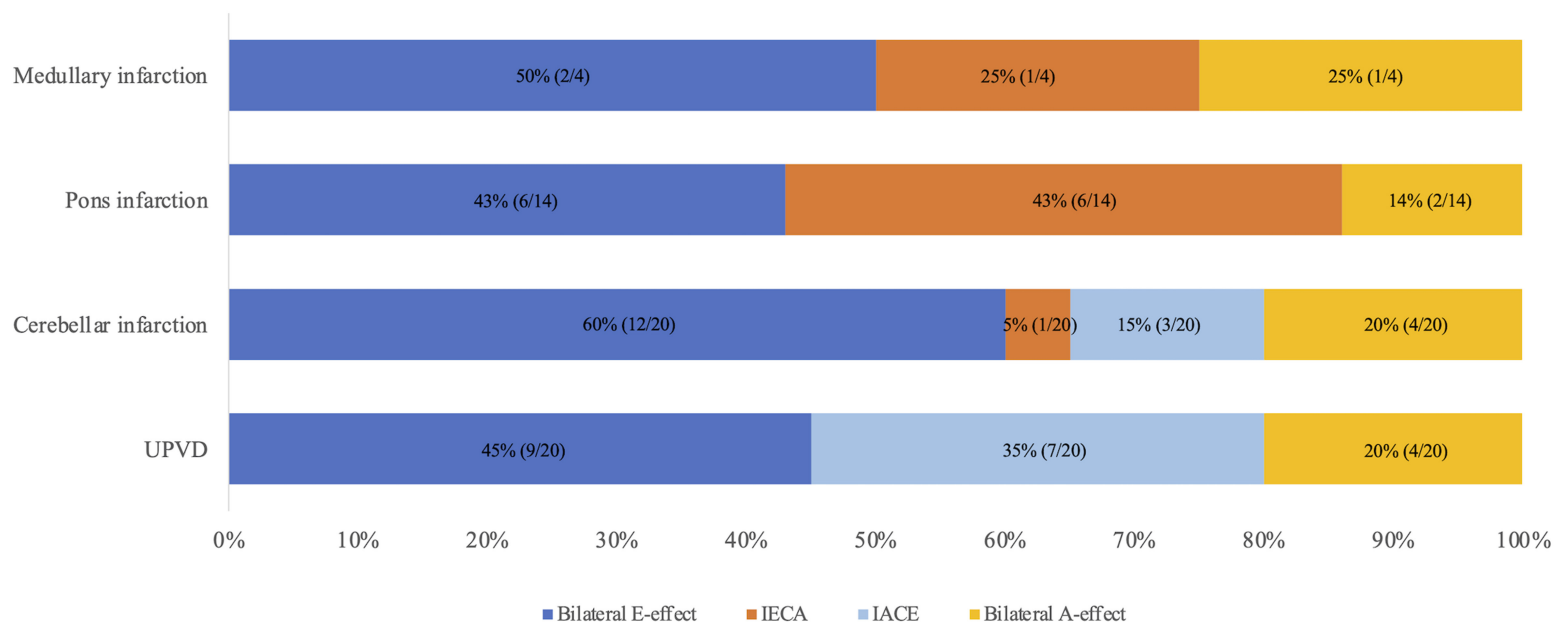
Distribution of A/E-effects of head tilt SVV (±45°) in medullary, pons, cerebellum, and UPVD groups. IECA, Ipsilesional E-effect contralesional A-effect; IACE, Ipsilesional A-effect contralesional E-effect; UPVD, unilateral peripheral vestibular disorders.

In the individuals with pontine infarction (*N* = 14), the HT-SVV errors on the ipsilateral side were −7.6 ± 10.8°, and that on the contralateral was 2.3 ± 14.9°. The incidence of bilateral E-effect, ipsiversive E-effect with contraversive A-effect, contraversive E-effect with ipsiversive A-effect, and bilateral A-effect were 43.0, 43.0, 0, and 14.0%, respectively. In the medullary infarction group (*N* = 4), the incidence of bilateral E-effect, ipsiversive E-effect with contraversive A-effect, contraversive E-effect with ipsiversive A-effect, and bilateral A-effect were 50, 25.0, 0, and 25.0%, respectively. In the CI group, the ipsilateral HT-SVV errors were −7.9 ± 12.2°, and that of the contralateral side was −8.2 ± 10.9°. The incidences of bilateral E-effect, ipsiversive E-effect with contraversive A-effect, contraversive E-effect with ipsiversive A-effect, and bilateral A-effect were 60, 5, 15, and 20%, respectively. In the UPVD group, the ipsilateral HT-SVV errors were 1.8 ± 9.6°, and that on the contralateral side was −8.0 ± 10.1°. The overall performance indicated the presence of the contraversive E-effect with ipsiversive A-effect. The incidences of bilateral E-effect, ipsiversive E-effect with contraversive A-effect, contraversive E-effect with ipsiversive A-effect, and bilateral A-effect were 45.0, 0, 35.0, and 20.0%, respectively. We compared the differences in the incidence of the A/E-effects of HT-SVV (±45°) in the ACVV and UPVD groups using the Chi-square test. We found that 35% of the patients with UPVD (7/20) showed ipsiversive A-effect and contraversive E-effect while 43% of the patients with pontine infarction (6/14) and 25% of patients with medullary infarction (1/4) had ipsiversive E-effect and contraversive A-effect. The incidence of the ipsiversive E-effect with contraversive A-effect in patients with pontine infarction was significantly higher than that in the UPVD group (χ^2^ = 13.68, *P* = 0.002, Pearson Chi-square test; Figure 5).

**Figure 6:**
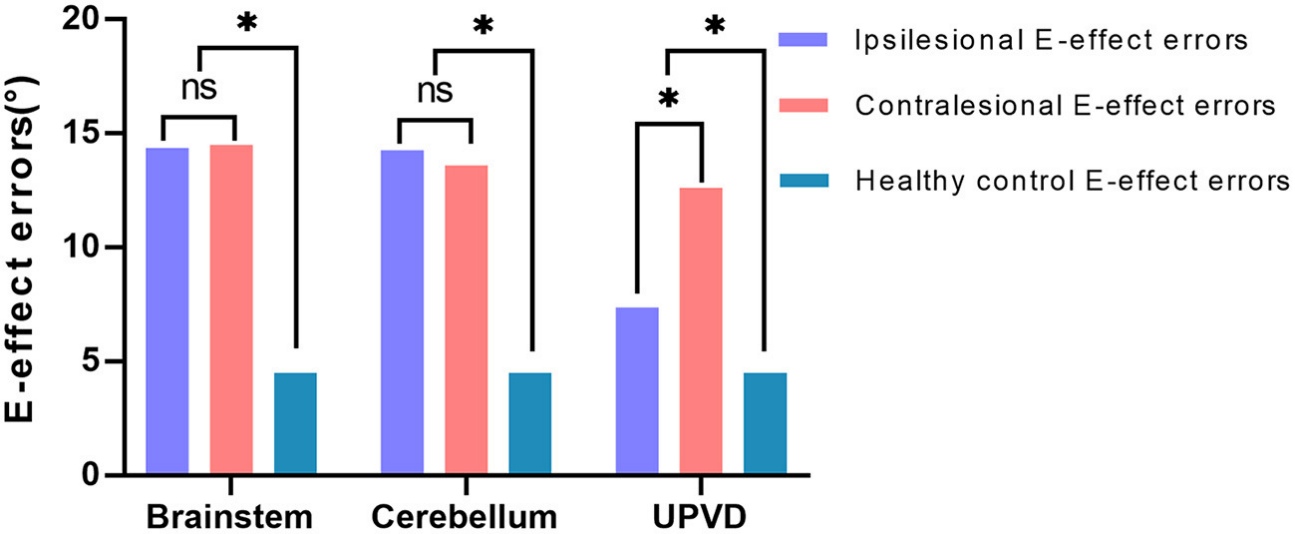
Difference analysis of the mean value of the bilateral E-effect in three patient groups. *P < 0.05. UPVD, unilateral peripheral vestibular disorders.

Since most of the ACVV (75%, 15/20) and some of the UPVD (45%, 9/20) patients exhibited bilateral E-effects, we further analyzed the absolute value and symmetry of the bilateral E-effect in these patients. Specifically, in patients and healthy controls, the symmetry of the E-effect of HT-SVV when the head tilted to both sides is the intra-group comparison. The absolute value of the E-effect of patients with head tilt to both sides was compared with the mean of the absolute value of the E-effect of the healthy control group between groups. In the healthy control group, the range of the E-effect was 0.1–9.5°, and the mean absolute value of the bilateral E-effect was 4.5 ± 2.1°. The absolute value of the bilateral E-effect in the three groups was significantly higher than that in the healthy control group. Among the groups, the absolute value of the ipsilesional E-effect in the UPVD group was significantly higher than that of the contralesional E-effect, indicating an asymmetric E-effect. However, we found no significant difference in the absolute value of the bilateral E-effect between the BI and CI groups, showing a symmetrical increase in the pathological E-effect (Figure 6 and Table 3).

**Table 3:** Absolute values and symmetry analysis of the bilateral E-effect according to injury.

	**Ipsilesional** **E-effect** **errors(°)**	**Contralesional** **E-effect** **errors(°)**	***t***	***P***
BI (*n* = 8)	14.38 ± 8.91**^a^**	14.51 ± 8.59**^a^**	0.194	0.852
*t*	3.152	2.982		
*P*	0.016	0.020		
CI (*n* = 12)	14.27 ± 10.84**^a^**	13.61 ± 9.81**^a^**	0.310	0.762
*t*	3.135	3.230		
*P*	0.009	0.008		
UPVD(*n* = 9)	7.38 ± 3.64**^a^**	12.63 ± 6.37**^a^**	2.391	**0.044** **^b^**
*t*	2.365	3.619		
*P*	0.041	0.006		

a There is a significant difference in the absolute value of the E-effect compared with the healthy control group.

b There is a significant difference in the absolute value of the E-effect between the ipsilesional and the contralesional sides. BI, brainstem infarction; CI, cerebellum infarction; UPVD, unilateral peripheral vestibular disorders.

The bold value means *P* < 0.05.

## Discussion

As with UPVD patients, we found that certain patients with ACVV, including that caused by LMI (100%, 2/2), pontine infarction (21%, 3/14), and cerebellar infarction (35%, 7/20), showed ipsiversive OTR. However, other patients with ACVV caused by MMI (50%, 1/2), partial pontine infarction (42%, 6/14), midbrain infarction (100%, 2/2), and cerebellar infarction (30.0%, 6/20) showed contraversive OTR. The graviceptive brainstem pathways originate from the vestibular nuclei (VN), cross the midline at the pontine level, and ascend in the medial longitudinal fascicle to the interstitial nucleus of Cajal (INC) in the midbrain. Therefore, lesions involving the medulla or those caudal to the pons before decussation cause ipsiversive OTR, whereas lesions affecting the region rostral to the pons and midbrain cause contraversive OTR (10). The direction of OTR is often uncertain in patients with lesions at the pontomedullary junction (20–22). There are several reasons for this. First, the precise anatomical localization of the level of the crossing of the graviceptive pathways has not been conducted (23). Second, medullary lesions, such as medial and lateral lesions of the medulla, involve different vestibular structures (VN, nucleus prepositus hypoglossi, inferior cerebellar peduncle, etc.) and thus lead to different OTR patterns (20).

Of the patients with ipsiversive OTR, 2 had lesions of the lateral medulla, and 3 had lesions of the caudal pons. Of the patients with contraversive OTR, 2 had lesions of the midbrain, and 6 had lesions of the rostral pons, which is consistent with the previous theories regarding OTR (10). However, 1 patient with MMI had a different OTR pattern compared with the patients with LMI, and showed isolated contraversive SVV tilt without HT, SD, and OT. Previous studies have shown that contraversive SVV tilt can be observed in patients with isolated MMI, which may indicate a unilateral lesion of the graviceptive brainstem pathways after decussation at the pontomedullary junction (24).

Of the 6 patients with pontine infarction showing contraversive OTR, only 1 (16%) had isolated SVV tilt, and 5 (83%) had at least 2 (including SVV tilt and OT) or more components of the OTR. Of the 4 patients (20%) with brainstem infarction showing a complete OTR, 2 had lesions of the lateral medulla with ipsiversive OTR, 1 had a lesion of the pons and showed contraversive OTR with internuclear ophthalmoplegia, and 1 had a lesion of the midbrain and showed contraversive OTR. Brandt et al. (10) examined patients with acute unilateral brainstem infarctions. They revealed that pathological tilts of SVV (94%) and ocular torsion (83%) were the most sensitive tests, such that only 20% of the patients showed complete OTR, consistent with our results.

Studies have shown that the OTR of patients with cerebellar lesions does not have a directionality according to the lesion side. There are three possible reasons for this. First, lesions involving the nodulus/uvula may lead to contraversive OTR because of a loss of inhibition over the ipsilesional VN (12, 25). Second, there are inhibitory projections from the dentate nucleus to the VN, and lesions of the dentate nucleus may lead to an increase in tonic resting activity in the ipsilesional VN because of a loss of inhibition. This could thus induce contraversive OTR (13). Third, as the lesions were mostly associated with the biventer lobule and inferior semilunar lobule when researchers observed ipsiversive signs of OTR, the disruption of inhibitory GABAergic efferents from the cerebellar cortex may enhance activity in the intact dentate nucleus and thus decrease tonic resting activity in the VN (13). This could lead to an ipsiversive OTR. Of the 6 patients with cerebellar infarcts showing contraversive OTR in our study, 1 had lesions of the unilateral nodulus/uvula and 2 had lesions of the dentate nucleus. This supports the hypothesis that lesions involving the dentate nucleus or nodulus/uvula may lead to disinhibition of the ipsilesional vestibular nuclear complex and result in contraversive OTR. The contraversive OTR observed in the 2 patients with large infarcts in the posterior inferior cerebellar artery (PICA) territory may be explained by damage to the afferent or efferent pathways to and from the dentate nucleus or nodulus/uvula (12, 13). We found that 1 patient with an isolated lesion in the posterior paravermis showed only contraversive SVV tilt. This suggests that midline structural components of the cerebellum other than the nodule/uvula and dentate nucleus may be involved in processing otolithic signals. Of the 7 patients with cerebellar infarction showing ipsiversive OTR in our study, 6 had lesions in the PICA territory, mostly involving the tonsils, biventer lobule, and inferior semilunar lobule, and 1 had a lesion of the middle cerebellar peduncle involving the anterior inferior cerebellar artery (AICA) territory. Hypoperfusion of the AICA might lead to infarction of its branch labyrinth artery, thus inducing ipsiversive OTR (26). Further studies are needed with a larger sample size to explore the precise localization of key cerebellar structures related to the OTR.

Recent studies have shown that SD is the only specific but non-sensitive (40%) sign of pseudoneuritis (27), and this has been incorporated into the bedside eye movement assessment (HINTS) conducted to differentiate between central and peripheral acute vestibular syndrome (28). Our study found that the frequency of SD was not high in ACVV patients. Instead, a minority of patients [brain stem infarction (25%, 5/25), cerebellar infarction (15%, 3/20), and UPVD (25%, 5/20)] had SD. Korda et al. (11) found that the SD prevalence was 24% in AUVP patients and 29% in stroke patients, consistent with our findings.

We used the monocular Maddox rod to examine SD and added a prism to correct vertical diplopia. In a previous study of 7 patients with acute vestibular neuronitis, all patients had a subtle 1 prism diopter hyperphoria that was only measurable with a Maddox rod test (15). This is consistent with our findings that UPVD patients have a small SD amplitude. The SD of the BI group was significantly greater than that of the UPVD group, and the AUC of the receiver operating characteristic curve corresponding to the use of SD to predict brainstem damage was 0.92, with a sensitivity of 100% and a specificity of 80% when SD ≥ 3°. In a previous study having adopted video-oculography to quantify SD, an SD greater than 3.3° corresponded to a specificity for predicting stroke of 98.1% and a sensitivity of 8.3%, similar to our findings (11). Patients with SD and central lesions have been found to exhibit impaired neural integration related to Listing's law, while there is no such related evidence for patients with peripheral lesions (29). This may be one of the reasons why the SD amplitude is larger in central lesion patients.

Our results indicate that SD has limited diagnostic value for patients with cerebellar lesions in ACVV. In a previous study of 27 patients with acute unilateral cerebellar infarction, no patients showed SD. In our study, only 3 patients (15%) with cerebellar infarcts showed SD, and the degree of SD was not different from that observed in UPVD patients. SD has been attributed to an afferent imbalance of utricular signals in oculomotor neurons through the brainstem or polysynaptic transmission through the cerebellum (30–32). Primary afferents arising from the utricle project to the second-order neurons in the VN, which then carry signals *via* the medial longitudinal fasciculus to the oculomotor and trochlear nuclei in the brainstem. The cerebellum also mediates the utricle-ocular reflex *via* a polysynaptic pathway, and primary utricular afferents have strong direct projections to the VN, cerebellar nodulus, and ventral uvula, with weaker projections to the anterior vermis, fastigial nuclei, and the flocculus and ventral paraflocculus (32, 33). There is an extensive projection network of otolithic signals within the cerebellum, and the cerebellum plays a critical role in sensorimotor signal transformation in the otolith-ocular pathway by computing an internal estimate of gravity (34, 35). Therefore, differences in SD in ACVV patients may be caused by differences in utricle-ocular reflex pathways involved in brainstem/cerebellar lesions.

SVV errors reflect challenges encountered by the brain in maintaining a common reference frame based on sensory information encoding eye, head, and body positions. Physiologically, SVV errors are biased toward the direction of the body position at tilt angles greater than 60° (known as the Aubert or A-effect). At tilt angles less than 60°, SVV errors are often biased in the opposite direction of the body position (known as the Müller or E-effect) (36). Otolithic inputs play a dominant role in the perception of verticality during small angle head tilts. Although prior studies have examined the mechanisms of the E- and A-effects, the origins of these effects are not well understood (18, 37).

In our study, we found that compared with the 35% of patients with UPVD (7/20) who showed an ipsiversive A-effect with a contraversive E-effect, among ACVV patients, the head tilt SVV (±45°) of 43% of the patients with pons infarction (6/14) and 25% of the patients with medulla infarction (1/4) exhibited the ipsiversive E-effect with a contraversive A-effect. The utricle responds to roll tilts and side-to-side translation of the head, and the hair cells for opposing polarization are aligned on either side of the striola. The hair cells are oriented toward the striola, with a 3:1 preponderance of the units with ipsilaterally directed vectors (18). The medial and lateral portions of the utricle respond differently according to the vectors generated by different degrees of head tilt. When the head is tilted in the direction of the axis of polarity of a hair cell unit, that cell depolarizes and excites the afferent vestibular fibers (18). During linear acceleration of the head, some hair cells are depolarized while others on the opposite side of the striola are hyperpolarized (cross-striolar inhibition) (38). Small angle head tilts mainly excite the lateral portion of the utricle and induce a small deflection in the hair cells in the opposite direction of head movements. This can contribute to the E-effect (18). When a patient with UPVD slightly tilts their head to the ipsilesional side, afferent signals from the lateral portion of the ipsilesional utricle cannot be generated, while simultaneous inhibition through the commissural inhibition of the utricle in the ipsilesional ear does not occur. Thus, the disinhibited neuronal activities from the lateral portion of the contraversive utricle deviate the SVV in the direction of the head tilt, resulting in the A-effect (18). In ACVV patients with pontine or medullary lesions, we observed a contralateral A-effect. This may have been caused by the afferent signals the crossed to the contralateral side from the primary utricle through the medial longitudinal fasciculus at the pontomedulla junction.

In this study, we found that a considerable proportion of UPVD (35%, 7/20) and ACVV patients exhibited bilaterally pathological E-effects. Interestingly, compared with healthy subjects, UPVD patients showed an asymmetric increase in the pathological E-effect, such that the absolute value of the contraversive E-effect was significantly higher than the ipsiversive one. In contrast, ACVV patients exhibited a symmetrical increase in the pathological E-effect. The asymmetric increase in the E-effect in UPVD patients might have been related to the loss of one utricle, such that the remaining one becomes bidirectionally sensitive (this process is thought to occur within 6–10 weeks). In this case, hypersensitivity of the contraversive utricle makes the absolute value of the contraversive E-effect higher than the ipsiversive one (39). Consistent with our findings, a chronic unilateral vestibular hypofunction study found that the adjustment errors of ±45° SVV was asymmetric, leading the authors to reject the hypothesis that a constant offset is added to physiological deviations in SVV adjustments when roll-tilting occurs (40). Tarnutzer et al. (41) examined 6 patients with central vestibular pathway lesions and compared SVV measurements in different roll orientations (0°, ±45°, and ±90°) in the subacute state (4–33 day). They found that two patients with cerebellar lesions exhibited a bilateral increased E-effect, consistent with our findings of bilateral symmetrical E-effects in patients with cerebellar lesions. Vestibulo-cerebellar lesion-induced loss of inhibitory function in the vestibular graviceptive pathway might have resulted in a bilateral E-effect because of overestimation of the direction of gravity. We also found that in ACVV patients with LMI, the direction of the OTR was similar to that for patients with peripheral lesions. However, the SD and ±45°SVV showed a central pattern.

This is presumably because the secondary neurons in the vestibular nucleus directly receive the primary afferents from the utricle but are also involved in the central modulation and integration of these signals. Thus, the lesions in our patients appear to be characterized by a mixed pattern of peripheral and central vestibular dysfunction.

## Limitations

There are some limitations to this study. First, we did not perform a follow-up assessment because of the cross-sectional design. Moreover, the sample size of this study was small, and it was a single-center study with the possibility of a selection bias. Further studies with a larger sample size are needed to explore the diagnostic value of OTR plus head tilt SVV (±45°) in ACVV patients.

## Conclusions

The evaluation of OTR plus head tilt SVV (±45°) in vertigo patients is helpful for identification and diagnosis of ACVV, especially when SD ≥ 3° and the the E-effects are symmetrically increased.

## Data availability statement

The original contributions presented in the study are included in the article/supplementary material, further inquiries can be directed to the corresponding authors.

## Ethics statement

The studies involving human participants were reviewed and approved by Ethics Committee of Aerospace Center Hospital, Peking University Aerospace School of Clinical Medicine. The patients/participants provided their written informed consent to participate in this study.

## Author contributions

XY designed the study. YF, TZ, YW, NS, and MZ performed the experiments and analyzed the data. YF and TZ drafted and prepared the manuscript. J-SK and XL corrected this manuscript. All authors read and approved the final manuscript.

## Funding

This study was supported by the Hygiene and Health Development Scientific Research Fostering Plan of Haidian District Beijing (HP2021-03-50703).

## Conflict of interest

The authors declare that the research was conducted in the absence of any commercial or financial relationships that could be construed as a potential conflict of interest.

## Publisher's note

All claims expressed in this article are solely those of the authors and do not necessarily represent those of their affiliated organizations, or those of the publisher, the editors and the reviewers. Any product that may be evaluated in this article, or claim that may be made by its manufacturer, is not guaranteed or endorsed by the publisher.
